# Mechanisms Governing Oligodendrocyte Viability in Multiple Sclerosis and Its Animal Models

**DOI:** 10.3390/cells13020116

**Published:** 2024-01-09

**Authors:** Zhixin Lei, Wensheng Lin

**Affiliations:** 1 School of Chemistry, Chemical Engineering and Life Science, Wuhan University of Technology, Wuhan 430070, China; leizhixin@whut.edu.cn; 2 Department of Neuroscience, University of Minnesota, Minneapolis, MN 55455, USA; 3 Institute for Translational Neuroscience, University of Minnesota, Minneapolis, MN 55455, USA

**Keywords:** multiple sclerosis, experimental autoimmune encephalomyelitis, oligodendrocyte, myelin, IFN-γ, oxidative stress, mitochondria, unfolded protein response, NF-κB

## Abstract

Multiple sclerosis (MS) is a chronic autoimmune inflammatory demyelinating disease of the central nervous system (CNS), which is triggered by an autoimmune assault targeting oligodendrocytes and myelin. Recent research indicates that the demise of oligodendrocytes due to an autoimmune attack contributes significantly to the pathogenesis of MS and its animal model experimental autoimmune encephalomyelitis (EAE). A key challenge in MS research lies in comprehending the mechanisms governing oligodendrocyte viability and devising therapeutic approaches to enhance oligodendrocyte survival. Here, we provide an overview of recent findings that highlight the contributions of oligodendrocyte death to the development of MS and EAE and summarize the current literature on the mechanisms governing oligodendrocyte viability in these diseases.

## 1. Introduction

Multiple sclerosis (MS) is an autoimmune inflammatory demyelinating disease of the central nervous system (CNS), driven primarily by T-cell-mediated inflammation [1,2,3,4]. The distinctive feature of MS pathology involves the occurrence of demyelinated plaques within the white matter of the CNS. These plaques are characterized by inflammation, the depletion of oligodendrocytes, demyelination, and the degeneration of axons. While the precise cause of MS remains uncertain, it is theorized to stem from an autoimmune assault targeting mature oligodendrocytes and the myelin sheath [1,2,3,4]. The prevailing theory suggests that the components of myelin induce the activation of T cells within the peripheral immune system of individuals with MS. Consequently, these T cells (with reactivity to myelin components) penetrate the blood–brain barrier, gaining entry into the CNS and instigating inflammation. In MS-related CNS inflammation, there is an infiltration of T cells, B cells, and monocytes, along with the activation of macrophages and microglia. This process leads to heightened levels of inflammatory cytokines and reactive oxygen species (ROS). The resulting inflammatory environment contributes to the demise of oligodendrocytes, myelin damage, and axon degeneration [1,2,3,4]. Genome-wide association studies (GWAS) have uncovered over 200 MS risk genes. A significant portion of these genes is linked to either autoimmunity or inflammation [1,2,3,4]. Experimental autoimmune encephalomyelitis (EAE) serves as the principal animal model for MS research, replicating numerous immunological, clinical, and pathological characteristics observed in MS [5,6,7,8]. In contrast to MS, which is a spontaneous and idiopathic disease, EAE is induced by inoculating animals, commonly mice and rats, with myelin components such as MOG (myelin oligodendrocyte glycoprotein), PLP (proteolipid protein), or MBP (myelin basic protein).

Oligodendrocytes play a crucial role in the CNS by producing the myelin sheath, which wraps around axons to provide insulation and protection [9,10]. In MS and EAE, oligodendrocytes face destruction from both specific, cell-selective immune mechanisms and non-specific bystander mechanisms [11,12,13]. Autoantibodies that target myelin components can trigger oligodendrocyte death and demyelination. This can occur through the activation of complement pathways or by binding to Fc-receptors on activated macrophages. Similarly, cytotoxic T cells, directed against myelin components, can induce oligodendrocyte death and demyelination. On the other hand, oligodendrocytes are particularly susceptible to ROS due to a combination of factors such as elevated intracellular iron, elevated metabolic rate, and reduced levels of antioxidant glutathione. Oxidative damage is a critical contributor to oligodendrocyte death in MS and EAE. Oligodendrocytes also express receptors that render them vulnerable to excitotoxic cell death. They carry kainate, AMPA, and NMDA receptors, making them susceptible to glutamate toxicity. Moreover, oligodendrocyte death can result from exposure to immune cytokines that are produced by myelin-reactive T cells. For instance, tumor necrosis factor α (TNFα) can cause oligodendrocyte apoptosis by binding to their p55 TNF receptor. Apart from these direct actions, immune cytokines have the potential to indirectly harm oligodendrocytes. This occurs through the activation of macrophages and microglia, leading to an upsurge in the production of ROS and inflammatory mediators [11,12,13].

Mounting evidence suggests that the death of oligodendrocytes, induced by autoimmune inflammation, significantly contributes to the development of MS and EAE [13,14]. The earliest structural alteration in newly developing demyelinating lesions in both MS and EAE is the apoptosis of oligodendrocytes, as observed in various studies [15,16]. Studies employing mice with an enforced expression of anti-apoptotic proteins (such as p35) or the knockout of pro-apoptotic proteins (such as the TNF receptor 1, Fas, and Fas-associated protein with death domain), specifically in oligodendrocytes, have demonstrated their protective effects on oligodendrocytes against inflammatory attacks. This protection leads to the amelioration of oligodendrocyte death, myelin damage, axon degeneration, and inflammation in the lesion sites and results in the attenuation of disease severity in the EAE model [17,18,19,20]. Several reports also showed that augmenting survival-signaling pathways in oligodendrocytes confers protection to mice against EAE, whereas impairing these pathways renders mice susceptible to the disease [16,21,22,23,24,25]. Notably, a study has shown that oligodendrocyte apoptosis alone is sufficient to trigger T cell-mediated autoimmunity against myelin, leading to immune-mediated CNS demyelination [26]. This highlights the pivotal role of oligodendrocyte death in pathogenies of MS and EAE.

Although considerable progress has been made in anti-inflammatory treatments for MS, there is no effective treatment that can enhance oligodendrocyte survival in MS [27,28,29]. A significant challenge in MS research lies in comprehending the mechanisms that dictate the viability of oligodendrocytes and in devising therapeutic strategies to shield these cells and myelin from inflammation. In this review, we summarize the current knowledge about the intrinsic mechanisms governing oligodendrocyte viability in MS and its animal models.

## 2. Mechanisms Regulating Oligodendrocyte Viability in MS and Its Animal Models

Evidence is accumulating that immune cytokines, oxidative stress, mitochondria, the unfolded protein response (UPR), and NF-κB signaling are the major players in regulating oligodendrocyte viability in MS and its animal models.

### 2.1. Immune Cytokines

Immune cytokines, including interferon-γ (IFN-γ) and TNFα, among others, are the key players in regulating the development of MS and EAE [1,2,3,4]. Due to the word limit, in the review, we focus on summarizing the effects of IFN-γ on oligodendrocytes in MS and its animal models. IFN-γ, a pleiotropic cytokine, intensifies inflammation by promoting the differentiation of Th1 T cells, activating microglia and macrophages, and inducing the expression of various inflammatory mediators. IFN-γ also influences oligodendrocyte viability in MS and its animal models [30,31,32]. IFN-γ applies its impact by attaching to its receptors on the cell surface, IFN-γR1 and IFN-γR2, triggering receptor oligomerization. This, in turn, activates Janus kinases 1 (JAK1) and JAK2, leading to the phosphorylation of both JAKs and the receptors [33,34]. Following receptor activation, the signal transducer and activator of transcription 1 (STAT1) is recruited and subsequently phosphorylated. Upon phosphorylation, STAT1 undergoes dimerization, moves to the nucleus, and regulates gene expression by attaching to gamma-activated sequence (GAS) elements found within the promoters of genes responsive to IFN-γ, including interferon regulatory factor 1 (IRF-1) [33,34]. On the other hand, suppressors of cytokine-signaling 1 (SOCS1) can inhibit IFN-γR-induced JAK/STAT1 signaling and modulate the impact of IFN-γ on cells [35].

IFN-γ, typically imperceptible in the healthy CNS, becomes detectable in the symptomatic phases of MS and EAE [31,36]. In vitro studies reveal that IFN-γ can directly act on oligodendrocytes to induce apoptosis [37,38]. However, the role of IFN-γ in MS and EAE is complex and sometimes contradictory. Administering IFN-γ to patients with MS and mice with EAE leads to enhanced inflammation in the CNS and exacerbated clinical symptoms [39,40,41]. Enforcing the expression of IFN-γ in the CNS induces inflammation and leads to abnormalities of oligodendrocytes and myelin in the CNS of transgenic mice [42,43]. Conversely, mice with a knockout of IFN-γ or its receptors maintain susceptibility to EAE, even developing the disease with increased morbidity and mortality [44,45]. Eliminating IFN-γ or its receptors renders mouse strains that are typically resistant to EAE susceptible to the disease [46]. CNS expression of IFN-γ before disease onset protects mice from EAE and prevents oligodendrocyte apoptosis and demyelination in the cuprizone model [47,48]. Moreover, there is evidence that the beneficial or detrimental effects of IFN-γ in EAE are dependent on the timing of its presence [49,50]. Enforced expression of IFN-γ in the CNS at the recovery stage of EAE hampers the process of recovering from the disease, which is accompanied by attenuated oligodendrocyte regeneration and remyelination in EAE demyelinated lesions [49]. In contrast, enforced expression of IFN-γ in the CNS before EAE onset ameliorates disease severity, which is accompanied by attenuated oligodendrocyte death, myelin damage, and axon damage in the CNS [50]. Additionally, these studies suggest that the paradoxical effects of IFN-γ on EAE development are mediated by the UPR [49,50]. Notably, a report demonstrated that IFN-γ exerts its effects on oligodendrocytes through JAK/STAT1 signaling in mouse models [51].

Furthermore, utilizing transgenic mice expressing SOCS1 exclusively in oligodendrocytes, a study demonstrated that enforced SOCS1 expression blocks oligodendrocyte response to IFN-γ, resulting in an acceleration of EAE development and an increase in EAE-induced oligodendrocyte apoptosis [21]. Intriguingly, GWAS has shown that IRF-1 is an MS risk gene [52]. IRF-1 knockout mice, while appearing normal under physiological conditions, exhibit resistance to EAE with abnormal IFN-γ responses [53]. Using transgenic mice expressing dominant-negative IRF-1 (dnIRF-1) exclusively in oligodendrocytes, a study showed that enforced expression of dnIRF-1 in oligodendrocytes attenuates disease severity and ameliorates oligodendrocyte apoptosis and myelin damage in the EAE model [22]. In summary, the available data collectively suggest that IFN-γ exerts its direct actions on oligodendrocytes in MS and EAE through IFN-γR-JAK/STAT-IRF-1 signaling, although its precise role in oligodendrocyte viability remains a subject of controversy.

### 2.2. Oxidative Stress

Oxidative stress stands as a prominent factor propelling tissue damage in inflammatory diseases, including MS and EAE [54,55]. This stress is primarily induced by the production of ROS, mainly by microglia and macrophages, during inflammation. Cells possess biological antioxidants, such as glutathione, ascorbic acid, and carotenoids, which interact with various oxidants to neutralize ROS and detoxify them. Nevertheless, when the production of ROS surpasses the cellular antioxidant capacity, elevated levels of ROS can induce the breakdown of essential cellular components, including lipids, proteins, and DNA, eventually culminating in cell apoptosis or necrosis [56,57]. Oligodendrocytes are highly susceptible to ROS because of reduced levels of antioxidant glutathione [58,59]. ROS can also disrupt the function of the mitochondrial respiratory chain, causing electron leakage and further contributing to oxidative injury [60]. Another factor that amplifies oxidative damage is the release of divalent iron from damaged cells into the extracellular space [61]. Moreover, oligodendrocytes express receptors for kainate, AMPA, and NMDA, making them susceptible to glutamate excitotoxicity in MS and EAE [62,63]. Glutamate excitotoxicity can trigger oligodendrocyte apoptosis by inducing mitochondrial depolarization and oxidative stress [62,63].

Cells possess an inherent mechanism designed to counteract excessive ROS and shield against oxidative damage. This mechanism, known as the oxidative stress response, is primarily regulated by the transcription factor nuclear factor (erythroid-derived 2)-like 2 (Nrf2) [64,65]. The modulation of Nrf2 activity primarily occurs at the protein level, regulated by its redox-sensitive inhibitor known as the kelch-like ECH-associated protein 1 (Keap1). In normal circumstances, Keap1 guides Nrf2 towards proteasomal degradation via polyubiquitination. However, when exposed to oxidants or electrophiles, crucial thiol groups within Keap1 undergo oxidation, leading to a conformational change in Keap1 and the subsequent liberation of Nrf2. Following this liberation, Nrf2 moves to the nucleus and attaches to antioxidant response elements (ARE) situated in the promoter region of various genes [64,65]. These genes encode proteins with cytoprotective functions, including heme oxygenase 1, NADPH quinone oxidoreductase, and sulfiredoxin, among others [66]. The activity of these enzymes proves effective in preventing cell damage associated with oxidative stress and inhibiting the initiation of apoptosis [64,65,66].

The level of Nrf2 is elevated in active MS lesions. The nuclear Nrf2 expression and upregulation of its downstream targets (such as heme oxygenase 1) are particularly strong in oligodendrocytes in MS lesions [67]. An in vitro study utilizing Nrf2 knockdown with shRNA demonstrated an exacerbation of oligodendrocyte apoptosis in response to oxidative stress. Conversely, Nrf2 activation achieved through the knockdown of its inhibitor, Keap1, showed an attenuation of oligodendrocyte apoptosis under oxidative stress conditions [68]. Several investigations have revealed that a global deficiency in Nrf2 exacerbates disease severity, demyelination, and inflammation in the EAE model [69,70]. On the other hand, compounds that activate Nrf2 signaling have been shown to reduce EAE disease severity. Notably, Dimethyl fumarate (DMF) induces modifications to thiol groups on the Nrf2 inhibitor Keap1, thereby stabilizing the Nrf2 protein and promoting the elevated expression of cytoprotective genes targeted by Nrf2, ultimately resulting in the attenuation of EAE disease severity [71,72,73]. Crucially, a study utilizing mice with Nrf2 deficiency exclusively in oligodendrocytes has shown that Nrf2 deficiency does not impact oligodendrocyte viability and function under normal, physiological conditions but exacerbates oligodendrocyte death and axonal degeneration in the cuprizone model [74]. Collectively, these findings underscore the crucial role of the Nrf2-mediated oxidative stress response in regulating oligodendrocyte viability in MS and its animal models.

### 2.3. Mitochondrial Damage

Mitochondria, which are double-membrane-bound subcellular organelles, are vital for fundamental cellular processes such as ATP production, calcium signaling, and iron homeostasis [75,76,77]. They face constant challenges from oxidative stress due to ROS generated in the electron transport chain [75,76,77,78]. Maintaining a proper mitochondrial function relies on a complex web of mitochondrial quality-control mechanisms, encompassing ROS scavenging, DNA repair, protein refolding/degradation, mitochondrial fusion and fission, mitophagy, and mitochondrial biogenesis [75,76,77,78]. Mitochondrial fusion promotes the mixing of content between healthy and partially dysfunctional mitochondria, while fission segregates damaged components [77,79]. Mitophagy selectively targets damaged or dysfunctional mitochondria for lysosomal degradation [77,80]. Mitochondria also have a central role in apoptosis. The release of cytochrome c from mitochondria, followed by caspase activation, represents a crucial mechanism through which mitochondrial damage induces cell apoptosis [81,82,83].

Mitochondrial dysfunction/damage in oligodendrocytes has been implicated in various demyelinating diseases [84,85,86]. Oligodendrocyte death and demyelination are observed in various inherited mitochondrial diseases in humans, such as Leber’s hereditary optic neuropathy (LHON) and Kearns–Sayre Syndrome [86,87]. LHON mutations also increase the risk of developing MS [88]. A study showed that double-strand breaks of mitochondrial DNA (mtDNA) in oligodendrocytes result in mitochondrial dysfunction, oligodendrocyte apoptosis, and demyelination in mice [89]. Data indicate that neurotoxin cuprizone causes oligodendrocyte apoptosis in the cuprizone-induced demyelination model by inducing mitochondrial dysfunction [90,91,92]. Notably, evidence suggests that inflammation-induced mitochondrial damage contributes to oligodendrocyte apoptosis in MS and EAE [84,85,86,93,94,95]. In vitro studies demonstrated that inflammatory mediators, including immune cytokines and ROS, induce oligodendrocyte apoptosis by damaging mitochondria [96,97,98]. Mitochondrial defects in oligodendrocytes are observed in acute MS lesions [93]. Utilizing a novel mouse model expressing the H_2_O_2_ biosensor mito-roGFP2-Orp1, researchers demonstrated significant mitochondrial oxidation in oligodendrocytes during EAE. Intriguingly, this redox change is apparent even prior to EAE onset, preceding CNS inflammation, and persists during the clinical phase, accompanied by reduced mitochondrial density and impaired mitochondrial morphology [94]. Another study revealed the activation of dynamin-related protein 1 (Drp1), a mitochondrial fission protein, in cultured oligodendrocytes treated with TNFα or ROS and in the EAE and cuprizone models [99]. Inhibiting Drp1 activation with its selective inhibitor P110 attenuates mitochondrial fragmentation and cell death in cultured oligodendrocytes treated with TNFα and ROS. Treatment with P110 attenuates disease severity, oligodendrocyte loss, and myelin damage in mice undergoing EAE, and it also reduces oligodendrocyte loss and myelin damage in the cuprizone model [99]. Conversely, another in vitro study showed that the mitochondrial division inhibitor 1 (mdivi-1) efficiently suppresses Drp1-mediated mitochondrial fission but does not alter oligodendrocyte viability during the lethal activation of AMPA receptors [100].

Furthermore, several studies suggest that mitophagy influences oligodendrocyte viability in demyelinating diseases. However, these studies are highly contradictory [95,101]. It has been shown that the concentrations of PARKIN and PINK1 (the master regulators of mitophagy) are elevated in the serum and cerebrospinal fluid of patients with MS [102,103]. A study showed that treatment with Haloperidol or Clozapine impairs mitophagy and attenuates demyelination in the cuprizone model [101]. In contrast, another study showed that treatment with Matrine enhances mitophagy and attenuates disease severity, oligodendrocyte loss, and demyelination in the EAE model [95]. Collectively, these data suggest the significant contribution of mitochondrial damage to oligodendrocyte apoptosis and the involvement of mitochondrial quality control mechanisms (especially mitochondrial fission and mitophagy) in regulating mitochondrial function and oligodendrocyte viability in MS and its animal models. However, the precise roles of mitochondrial fission and mitophagy in oligodendrocytes in MS and EAE remain elusive and warrant further investigation.

### 2.4. The UPR

**Figure 1:**
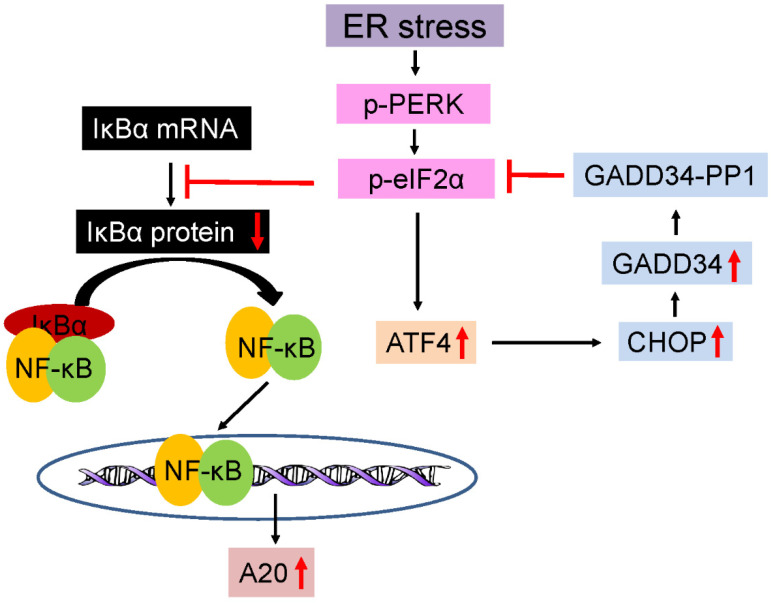
Schematic diagram of the PERK-eIF2α-NF-κB-A20 pathway. ER stress initiates PERK phosphorylation, leading to phosphorylation of eIF2α. P-eIF2α facilitates the translation of ATF4. ATF4, in turn, stimulates the expression of CHOP. CHOP induction downregulates the PERK-eIF2α pathway by increasing the expression of GADD34, which forms a complex with PP1, known as the GADD34-PP1 complex, to dephosphorylate p-eIF2α. P-eIF2α also triggers the activation of NF-κB pathway by inhibiting the translation of IκBα. NF-κB activation can stimulate the expression of multiple anti-apoptotic proteins, including A20/TNFAIP3.

The endoplasmic reticulum (ER) serves as the hub for the modification and folding of membrane and secretory proteins in eukaryotic cells. Disruptions in protein modification or folding cause the buildup of unfolded or misfolded proteins within the ER, resulting in ER stress and the activation of ER stress sensors, including inositol requiring enzyme 1 (IRE1), pancreatic ER kinase (PERK), and activating transcription factor 6 (ATF6). The activation of these three ER stress sensors coordinates an adaptive program known as the UPR [104,105,106]. The activation of PERK inhibits global protein translation but promotes the translation of the transcription factor ATF4 by phosphorylating eukaryotic translation initiation factor 2α (eIF2α). ATF4, in turn, enhances the expression of various stress-responsive genes, including cytoprotective genes and CHOP (CAATT enhancer–binding protein homologous protein). The upregulation of CHOP downregulates the activity of the PERK-eIF2α pathway by increasing the expression of GADD34 (growth arrest and DNA damage 34), which, along with PP1 (protein phosphatase 1), dephosphorylates p-eIF2α (phosphorylated eIF2α), establishing a robust negative feedback loop (Figure 1). The activation of IRE1 causes the splicing of X-box binding protein 1 (XBP1) mRNA, inducing the expression of stress-responsive genes. ATF6 becomes active as it translocates to the Golgi complex, where proteases S1P and S2P cleave it. The cleaved ATF6 then moves to the nucleus, acting as a transcription factor that stimulates the expression of stress-responsive genes. The principal goal of the UPR is to reinstate ER homeostasis and facilitate cellular adaptation to challenging conditions [104,105,106]. Nevertheless, in cases where adaptive measures fall short, the UPR activates apoptosis programs as a means to eliminate stressed cells. [107,108]. Recent studies revealed the UPR activation in oligodendrocytes in MS and EAE [109,110,111]. Significantly, a substantial body of research suggests that the UPR plays a crucial role in regulating the viability of oligodendrocytes in these diseases [111,112,113].

Several studies have highlighted the protective role of the PERK-eIF2α pathway in safeguarding oligodendrocytes and myelin from inflammation during EAE [111,112,113]. A study reported that CNS-specific IFN-γ expression prior to EAE onset leads to a reduction in EAE severity and alleviation of EAE-induced oligodendrocyte apoptosis, demyelination, and axon degeneration [50]. The protective effects of IFN-γ in EAE are linked to PERK activation in oligodendrocytes, and they are completely abrogated by global PERK heterozygous knockout [50]. Previous reports also demonstrated that mice with PERK knockout, specifically in oligodendrocytes, exhibit increased EAE severity, along with heightened oligodendrocyte loss, myelin damage, and axon degeneration [23,25]. Moreover, a mouse model featuring the controllable activation of the PERK pathway, specifically in oligodendrocytes, termed *PLP/Fv2E PERK* mice, was generated [16]. These mice express Fv2E-PERK, an engineered PERK variant controlled by the dimerizer AP20187 and independent of ER stress, exclusively in oligodendrocytes [16]. The administration of a low dose of AP20187 moderately activates the PERK-eIF2α pathway, specifically in oligodendrocytes, without impacting their viability or function under normal conditions. Strikingly, the moderate activation of PERK, specifically in oligodendrocytes, initiated before EAE onset, mitigates disease severity and attenuates oligodendrocyte apoptosis, myelin damage, and axon degeneration in the EAE model [16]. Additionally, earlier research has also proposed the therapeutic promise of selective inhibitors targeting GADD34, Guanabenz and Sephin1, in MS. Treatment with these inhibitors increases p-eIF2α levels in oligodendrocytes, resulting in decreased disease severity and an improvement in oligodendrocyte survival and myelin damage in the EAE model [114,115]. Collectively, these findings underscore the protective role of the PERK-eIF2α pathway in oligodendrocytes in MS and EAE.

Furthermore, recent investigations propose that the activation of the PERK-eIF2α pathway promotes oligodendrocyte survival in MS and EAE by activating the NF-κB pathway [25]. ATF4 is recognized as the principal transcription factor of the PERK-eIF2α pathway [104,105,106]. However, a recent study revealed that ATF4 knockout selectively in oligodendrocytes does not affect EAE severity or the associated oligodendrocyte death, myelin damage, or axon degeneration, despite the observed activation of ATF4 in oligodendrocytes in the EAE model [116]. Consistent with this, a prior study demonstrated that the global knockout of CHOP, a major ATF4-target gene, has a minimal effect on EAE development [117]. Hence, it appears unlikely that ATF4 participates in the beneficial role of the PERK-eIF2α pathway in oligodendrocytes in the EAE model. Conversely, the PERK-eIF2α pathway has the capability to activate NF-κB signaling by suppressing the translation of its inhibitor IκBα (Figure 1) [118]. Studies conducted both in vitro and in vivo have shown that the activation of the PERK-eIF2α pathway results in the activation of NF-κB signaling in oligodendrocytes. [16,119]. Both in vitro and in vivo studies have also suggested that the activation of NF-κB signaling protects oligodendrocytes against inflammation [24,119,120]. Importantly, a recent investigation revealed that heightened NF-κB activation selectively in oligodendrocytes fully counteracts the detrimental impacts of PERK knockout in oligodendrocytes during EAE [25]. These data suggest that the activation of NF-κB serves as the fundamental mechanism accountable for the beneficial role of the PERK-eIF2α pathway in oligodendrocytes in MS and EAE (Figure 1).

While one report showed that the IRE1 pathway is activated in MS lesions [109], there is no evidence that this pathway is involved in modulating oligodendrocyte function or viability under physiological and pathological conditions [113,121,122]. Conversely, recent research proposes a role for the ATF6α pathway in MS and EAE. A report showed that global ATF6α knockout increases disease severity and facilitates oligodendrocyte death and demyelination, but it does not impact inflammation in the EAE model [123]. Interestingly, the detrimental effects of ATF6α knockout observed in the EAE model are linked to impaired expression of BiP (immunoglobulin heavy chain-binding protein), a major ATF6α-target gene, in oligodendrocytes [123]. Consistent with these findings, another study demonstrated that mice with heterozygous BiP knockout exclusively in oligodendrocytes exhibit worsened disease severity and exacerbated oligodendrocyte loss and demyelination during EAE [121]. Consequently, these findings indicate that the ATF6α-BiP pathway exerts protective effects on oligodendrocytes in MS and EAE.

### 2.5. NF-κB Signaling

The transcription factor NF-κB plays a critical role in modulating inflammation and cell viability in inflammatory diseases like MS and EAE [120,124,125]. It manifests as a heterodimer or homodimer within the Rel family, encompassing p65, c-Rel, RelB, p50, and p52 [126,127]. In its inactive state, NF-κB is confined to the cytoplasm by interacting with its inhibitors (IκBs). Upon activation, NF-κB separates from IκBs and relocates to the nucleus, where it binds to the κB consensus DNA sequence, triggering the transcription of genes associated with inflammation and cell viability. NF-κB can be activated by various pathways, including the IκB kinase 2 (IKK2)-dependent canonical pathway, the noncanonical pathway, and atypical pathways [120,127,128]. The canonical pathway is triggered when cell surface receptors bind to their ligands (such as TNF receptors binding with TNFα), leading to the formation of the IKK complex, composed of IKK1, IKK2, and NEMO. This complex phosphorylates IκBα, resulting in its polyubiquitination and rapid degradation by the proteasome. Subsequently, NF-κB is released [126,127]. Atypical pathways, which are IKK2-independent but IκBα dependent, can activate NF-κB through mechanisms that reduce the level of IκBα [120,127,128]. The NF-κB pathway is further regulated by many negative feedback regulators, including A20/TNFAIP3, IκBα, Cezanne, and CYLD, among others [129]. A20/TNFAIP3 can attenuate NF-κB activation by blocking the canonical pathway [129]. IκBα can attenuate NF-κB activation by blocking both the canonical pathway and atypical pathways [129].

The NF-κB pathway is activated in both inflammatory cells and oligodendrocytes in MS and EAE [16,130,131]. While it is well-documented that the activation of the NF-κB pathway in inflammatory cells, such as T cells and monocytes, contributes to the development of MS and EAE by fostering inflammation [120,125,132,133,134], there is intriguing evidence suggesting a protective role for NF-κB activation in oligodendrocytes against inflammation in these conditions [120]. Numerous in vitro studies showed that the activation of the NF-κB pathway enhances the survival of oligodendrocytes exposed to inflammatory mediators [119,135,136,137]. Another in vitro study showed that the enhanced expression of cellular FLICE (FADD-like IL-1β-converting enzyme)-inhibitory protein (cFLIP), an NF-κB-target gene, safeguards oligodendrocytes from the cytotoxicity of TNFα [138]. An in vivo study employing mice expressing IκBαΔN (a deletion mutant, devoid of the N-terminal 36 amino acids of IκBα, acts as a dominant suppressor of NF-κB signaling [139]), selectively in oligodendrocytes revealed that the enforced expression of IκBαΔN impedes the activation of NF-κB signaling in oligodendrocytes and makes (re)myelinating oligodendrocytes more susceptible to the cytotoxicity of IFN-γ in young, developing mice as well as in the cuprizone-induced demyelination/remyelination model [24]. Blocking NF-κB activation in oligodendrocytes via the enforced expression of IκBαΔN also results in very severe EAE disease severity without affecting inflammation [24]. Moreover, using mice with enforced expresses a low level of IKK2ca (a constitutively active form of IKK2), specifically in oligodendrocytes. A study reported that the enforced expression of a low level of IKK2ca leads to the mild activation of NF-κB signaling in oligodendrocytes, and that the mild activation of NF-κB signaling in oligodendrocytes does not impact oligodendrocyte differentiation or viability under normal, physiological conditions but mitigates disease severity, oligodendrocyte loss, and myelin damage in the EAE model [25]. Additionally, a study reported that the sensitivity of oligodendrocytes to inflammation is heightened when cFLIP is knocked down using a virus expressing cFLIP-specific shRNA [138]. In contrast, a study reported that IKK2 knockout exclusively in oligodendrocytes has a minimal impact on EAE development [140]. Another study showed that the long-term expression of a high level of IKK2ca, specifically in oligodendrocytes, causes the strong activation of NF-κB signaling and results in neuroinflammation and myelin abnormalities in the CNS of adult mice [141]. These data suggest that the beneficial or detrimental effects of NF-κB activation on oligodendrocytes are dose-dependent and/or context-dependent.

Evidence suggests that the IKK2-dependent canonical pathway can activate NF-κB in oligodendrocytes in MS and EAE [125,142]. Recent studies demonstrated that the activation of the PERK-eIF2α pathway in response to ER stress triggers the activation of NF-κB signaling in oligodendrocytes by repressing the translation of IκBα (an atypical pathway) (Figure 1) [16,25,119]. Interestingly, evidence suggests that the atypical pathways, instead of the IKK2-dependent canonical pathway, are the major contributors to the cytoprotective effects of NF-κB activation on oligodendrocytes in MS and EAE [16,25,140]. Oligodendrocyte-specific IKK2 knockout (blockage of the IKK2-dependent canonical NF-κB pathway) does not affect EAE development [140]. Conversely, the oligodendrocyte-specific expression of IκBαΔN (blockage of both the IKK2-dependent canonical NF-κB pathway and atypical NF-κB pathways) dramatically exacerbates EAE disease severity [24]. The oligodendrocyte-specific expression of IKKca, which activates NF-κB without the involvement of its upstream pathways (including the canonical pathway, the non-canonical pathway, or atypical pathways), mildly activates NF-κB signaling and results in attenuated oligodendrocyte death in the EAE model [25]. Oligodendrocyte-specific PERK knockout diminishes NF-κB activation and exacerbates oligodendrocyte death in the EAE model [25]. Importantly, mild NF-κB activation caused by IKKca expression specifically in oligodendrocytes completely rescues the adverse effects of PERK knockout on oligodendrocytes in the EAE model [25]. These data suggest the protective effects of the PERK-eIF2α -NF-κB pathway on oligodendrocytes in MS and EAE (Figure 1).

NF-κB is recognized for its cytoprotective role by inducing specific anti-apoptotic genes, such as A20/TNFAIP3, cIAPs, cFLIP, Bcl-2, and/or Bcl-xL (Figure 1) [127,143,144]. However, the precise mechanisms through which NF-κB activation safeguards oligodendrocytes against inflammation in MS and EAE remain elusive. A recent study, employing whole-genome RNA sequencing, revealed that mild NF-κB activation induced by IKKca expression, specifically in oligodendrocytes, significantly upregulated only 12 genes. Among these, A20/TNFAIP3, an NF-κB-target, anti-apoptotic gene, stood out [25]. This study further illustrated that NF-κB activation increases A20/TNFAIP3 expression under normal conditions and during EAE. Moreover, this study showed that the detrimental effects of PERK knockout on oligodendrocytes in the EAE model are accompanied by impaired A20/TNFAIP3 expression in oligodendrocytes [25]. These results propose the intriguing notion that A20/TNFAIP3 may mediate the protective effects of NF-κB activation on oligodendrocytes in MS and EAE (Figure 1). Importantly, recent investigations have linked polymorphisms in TNFAIP3 (encoding the A20 protein), associated with a reduced function or expression of A20, with an increased susceptibility to MS [145,146,147]. Despite these observations, the role of A20/TNFAIP3 in oligodendrocytes in MS and its animal models has not been explored. A thorough examination of the role of A20/TNFAIP3 in oligodendrocytes and its impact on the protective effects of NF-κB activation on oligodendrocytes in animal models of MS is justified. 

## 3. Therapeutic Potential and Future Directions

Growing evidence indicates that the death of oligodendrocytes caused by inflammation significantly contributes to the development of MS. To impede disease progression in MS patients, it is imperative to explore therapeutic approaches that safeguard oligodendrocytes from inflammation. Despite notable advancements in anti-inflammatory treatments for MS, there remains a critical gap in the absence of an effective intervention to mitigate oligodendrocyte death and demyelination. A primary hurdle in MS research involves comprehending the mechanisms dictating oligodendrocyte viability and formulating therapeutic strategies to shield these cells and preserve myelin in the face of inflammation.

Accumulating evidence suggests that targeting the Nrf2-mediated oxidative stress response holds promise as a therapeutic strategy for a range of diseases, including MS [148,149]. Several small chemical compounds that selectively influence the activity of the Nrf2-mediated oxidative stress response have been identified, including DMF, SF (isothiocyanate), CDDO-Me (triterpenoid), and RTA 408 (triterpenoid). Several of these compounds have advanced to clinical trials for treating various diseases. Notably, DMF has received approval for use in the treatment of MS [148,149,150]. Significant advancements have been achieved in the identification of small chemical compounds that specifically modulate the activity of the three individual pathways of the UPR [113,151]. Notably, Salubrinal, identified as a selective inhibitor of phosphatase complexes accountable for the dephosphorylation of p-eIF2α [152], and Guanabenz and Sephin1, which bind selectively to GADD34 and hinder the activity of the GADD34/PP1 complex, consequently diminishing the dephosphorylation of p-eIF2α [153,154], are among the compounds showing promise. Multiple studies have suggested the therapeutic potential of Salubrinal, Guanabenz, and Sephin1 in the context of MS [114,115,155]. Moreover, significant strides have been achieved in the discovery of small chemical compounds that mitigate mitochondrial damage [156]. Notably, Urolithin A, identified as a mitophagy activator, has demonstrated the ability to reduce mitochondrial damage and enhance cell survival across various disease models [157,158]. A recent study has suggested the therapeutic potential of Matrine, another mitophagy activator, in the context of MS [95]. While the development of small chemical compounds targeting Nrf2-mediated oxidative stress response, the UPR, and the maintenance of mitochondrial homeostasis holds promise for therapeutic advancement, effectively modulating these biological processes for MS treatment without inducing adverse effects poses a formidable challenge.

The current available data highlight the crucial roles played by IFN-γ signaling, oxidative stress, mitochondrial damage, the UPR, and NF-κB signaling in influencing oligodendrocyte viability in MS. However, their precise roles and underlying mechanisms remain unclear and necessitate further investigation. Conversely, it is established that oligodendrocyte progenitor cells (OPCs) possess the ability to proliferate and differentiate into remyelinating oligodendrocytes responsible for repairing myelin damage in demyelinated lesions in MS. Despite this regenerative potential, MS exhibits insufficient oligodendrocyte regeneration and remyelination, leading to the accumulation of unrepaired lesions and a progressive decline in neurological function [159,160,161,162]. Evidence suggests that remyelinating oligodendrocytes face increased vulnerability to various insults compared to mature oligodendrocytes due to the substantial production demands for myelin proteins and lipids necessary for assembling myelin sheaths, coupled with their elevated metabolic rate [9,111,113]. It is imperative to comprehend the mechanisms dictating the viability of remyelinating oligodendrocytes in MS demyelinating lesions, which may or may not share similarities with the mechanisms discussed in this review.

## 4. Conclusions

In this review, we summarize the current literature on the intrinsic mechanisms governing oligodendrocyte viability in MS and its animal models and discuss the therapeutic potential of targeting these mechanisms.
